# Impact of a short training on the recognition of excessively deep chest compressions during video-assisted cardiopulmonary resuscitation: a randomized controlled simulation trial

**DOI:** 10.1186/s12909-025-07524-w

**Published:** 2025-07-11

**Authors:** Jonas Paul Schulte, Martin Klasen, Michael Schauwinhold, Jörg Christian Brokmann, Christopher Plata

**Affiliations:** 1 Department for Acute and Emergency Medicine, Medical Faculty, RWTH Aachen University, Pauwelsstrasse 30, 52074 Aachen, Germany; 2 Department of Anesthesiology, Medical Faculty, RWTH Aachen University, Pauwelsstrasse 30, 52074 Aachen, Germany; 3 Institute for Medical Informatics, Medical Faculty, RWTH Aachen University, Pauwelsstrasse 30, 52074 Aachen, Germany

**Keywords:** V-CPR, Video-CPR, Video-assisted cardiopulmonary resuscitation, VA-CPR, Layperson CPR, Bystander CPR, Resuscitation

## Abstract

**Introduction:**

The early commencement of effective resuscitation is of fundamental importance for the survival of individuals experiencing out-of-hospital cardiac arrest. In such circumstances, video-assisted guidance by the dispatcher has been demonstrated to be advantageous for the recognition of cardiac arrest and the improvement of CPR quality. The present study investigates the effectiveness of a brief training program for control center dispatchers in the recognition of common errors during simulated resuscitation.

**Methods:**

The study was approved by the local ethics committee and registered at the German Clinical Trial Register on 27th of February 2024 (Registration number: DRKS00032661) prior to inclusion of the first participant. Within a two-armed group study design, paramedics and emergency physicians were randomly assigned to either an experimental group (*n* = 44) or a control group (*n* = 44). The experimental group was initially exposed to a targeted brief training aimed at enhancing the recognition of excessively deep chest compression depth. All participants evaluated 42 distinct video sequences showing seven typical errors during CPR. The shown CPRs were simulated on a training manikin and the videos were evaluated by the participants under laboratory conditions. The primary endpoint was the accurate evaluation of the presented videos with too deep compression depth in a laboratory setting.

**Results:**

A total of 3696 video sequences were evaluated. The experimental group demonstrated a significantly higher recognition rate for too deep chest compressions compared to the control group (87.9% vs. 59.2%, *p* < 0.001). With regard to the remaining errors, no significant differences were observed between the study groups. Overall, 2861 of the 3696 videos (77.7%) were correctly classified. The proportion of correctly classified videos was significantly higher in the experimental group compared to the control group (79.8% vs. 75.6%, *p* = 0.003), indicating a statistically significant effect of the intervention.

**Conclusion:**

The identification of chest compressions with too deep compression depth in a video of simulated CPR was found to increase significantly in evaluators who had undergone a brief training course.

**Trial registration:**

The trial was registered in the German Clinical Trial Register “BfArM - Deutsches Register Klinischer Studien (DRKS)” under the registration number DRKS00032661.

**Supplementary Information:**

The online version contains supplementary material available at 10.1186/s12909-025-07524-w.

## Introduction

Sudden cardiac arrest is one of the leading causes of death with 15 to 20% of deaths in western countries [1, 2]. Despite various efforts in research and progress in clinical emergency management, survival from sudden cardiac arrest remains low at of little more than 10% [3–5]. Early initiation of high quality chest compressions is crucial for survival and a good neurological outcome after out-of-hospital cardiac arrest (OHCA) [6–8]. Since laypersons are often overstrained with providing sufficient chest compressions, telephone-assisted CPR (T-CPR) has been implemented sufficiently in daily practice in rescue centers in 80% of European countries [9–11], showing various positive effects on patients’ survival [12–14]. Video-assisted cardiopulmonary resuscitation (VA-CPR) represents a novel approach to assist laypersons during resuscitation efforts and to ensure high quality chest compressions. The usability of this new approach has been evaluated recently, and positive effects on CPR quality were observed [15–17]. Consequently, the audiovisual real-time connection was identified as a valuable tool for assessing chest compression quality [18]. Thereby, video-based communication facilitates the acquisition of additional information by the emergency services operator from the scene of the incident. The live visual data can assist the operator in the recognition of critical situations and the provision of feedback during CPR. However, there is a gap in the literature regarding the structural requirements for the implementation of video-assisted CPR in practice. For instance, there is no information about the training required for dispatchers to identify common errors during CPR in a video sequence. Recently published studies demonstrated a low level of recognition of chest compression with too deep compression depth during video analysis [19, 20]. In Germany, dispatch centers are primarily staffed by certified paramedics. In addition, physician-supported telemedicine systems are increasingly employed to deliver video-based medical guidance, positioning both paramedics and emergency physicians as key groups for delivering instructions in video-assisted CPR.

We thus investigated the primary hypothesis, that a preceding training with video sequences of too deep chest compressions increases the rate of correct evaluation of video sequences showing this error. As a secondary endpoint, the effect of the training on other typical mistakes during chest compressions or correctly performed chest compressions was investigated. Finally, participants’ attitude towards VA-CPR was evaluated.

## Methods

### Study design and setting

The study was approved by the Ethics Committee of the University Hospital RWTH Aachen (Approval number: EK 24–041, 22nd of February 2024) and was registered at the German Clinical Trial Register prior inclusion of the first participant (Registration number: DRKS00032661). During this randomized, controlled simulation study, 88 participants evaluated 3696 video sequences showing a cardiopulmonary resuscitation on a manikin (AmbuMan^®^ Advanced, Ambu A/S, Ballerup, Denmark). Depending on the study group, participants received standardized training before the actual evaluation process. Reporting was carried out in accordance with the CONSORT reporting guidelines [21]. The study was financed by the Centre for Clinical Acute and Emergency Medicine’s own funds (University Hospital Aachen).

### Recruitment

We randomly recruited emergency medicine professionals by face-to-face interactions on the campus of the University Hospital RWTH Aachen, at Malteser Rescue Center Aachen, Aachen, Germany and Malteser Rescue Center Simmerath, Simmerath, Germany, Aachen School for Firefighters, Aachen, Germany and at the Main Department of the Fire Brigade of Aachen, Aachen Germany. All participants were informed about the general objective of the study, which was to explore the potential usefulness of video-assisted resuscitation in future applications. However, they were not informed that the specific aim of the training was to improve the recognition of excessively deep chest compressions. Data privacy for all participants was obtained and only anonymized data was used for further analyses.

### Inclusion and exclusion criteria

Participants had to be either emergency physicians or paramedics working in the rescue service with a minimum age of 18 and a maximum age of 68 years. An active role as a dispatcher was not an inclusion criterion for this study. In addition to refusal, we excluded participants who had already participated in a video-CPR trial.

### Randomization

**Fig. 1:**
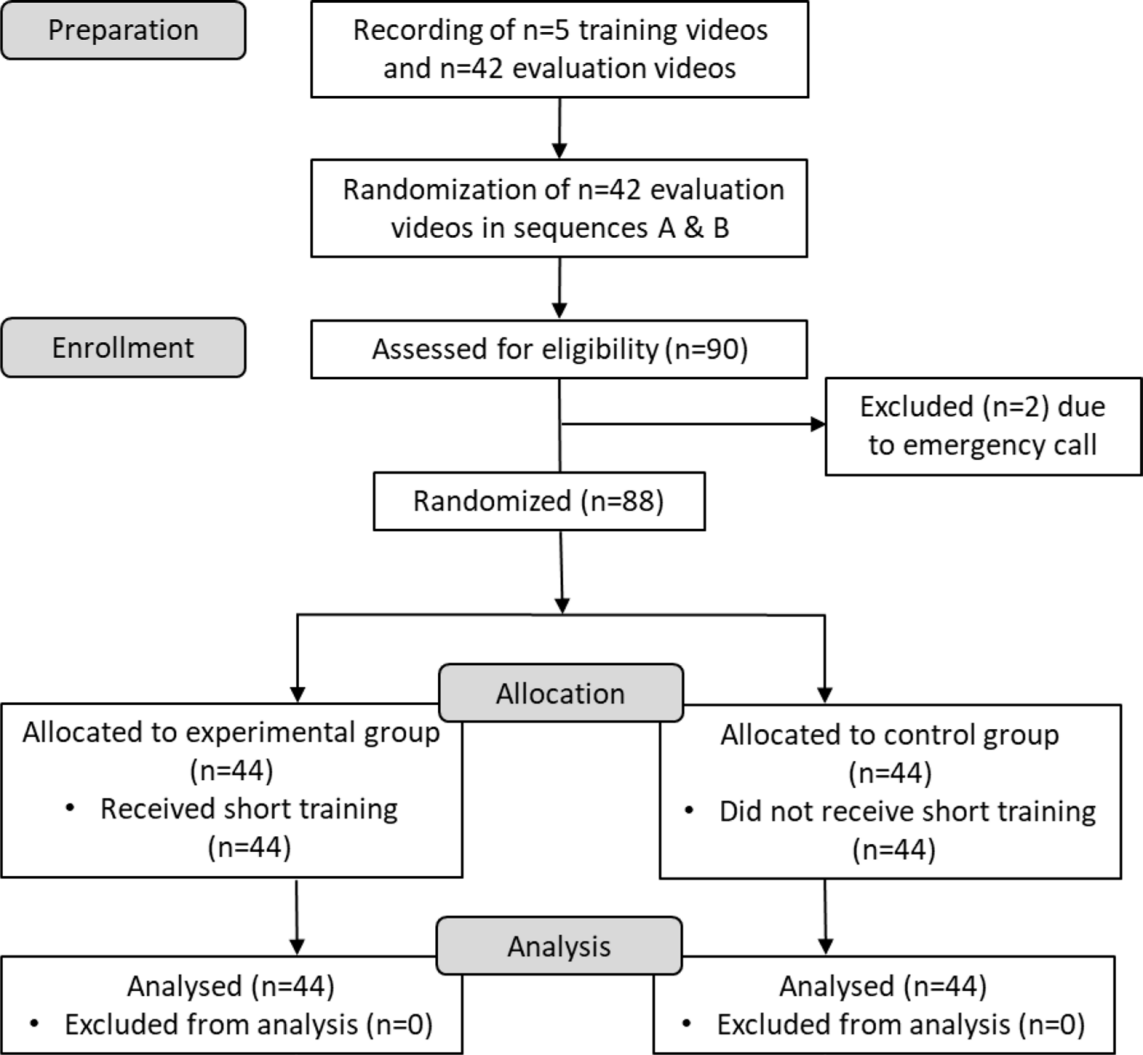
Study flow chart; EG: experimental group, CG: control group

The participants were randomly assigned to one of two study groups: the control group (which did not receive any training), and the training group. A randomization list, containing the numbers 1 through 88, was generated by a member of the study team using the randomization function in MS Excel (Microsoft Office, Excel, Version 2016). This team member was not involved in the subsequent enrollment process. Subsequently, the randomization numbers for each participant were incorporated into the preparation of lots. Following their consent to participate in the experiment, each subject was requested to draw a lot in order to be assigned to a study group. The order of the 42 video sequences employed during the evaluation process was randomized and the videos were compiled into two versions, designated A and B. For version B, the order of the videos from version A was reversed. Participants of both study groups were assigned to either version A or B using the “Coin Flip App 1.0” (Coin flip app for Iphone by Sebastian Kruger via App Store). Both versions were utilized equally in both study groups (see Fig. 1).

**Fig. 2:**
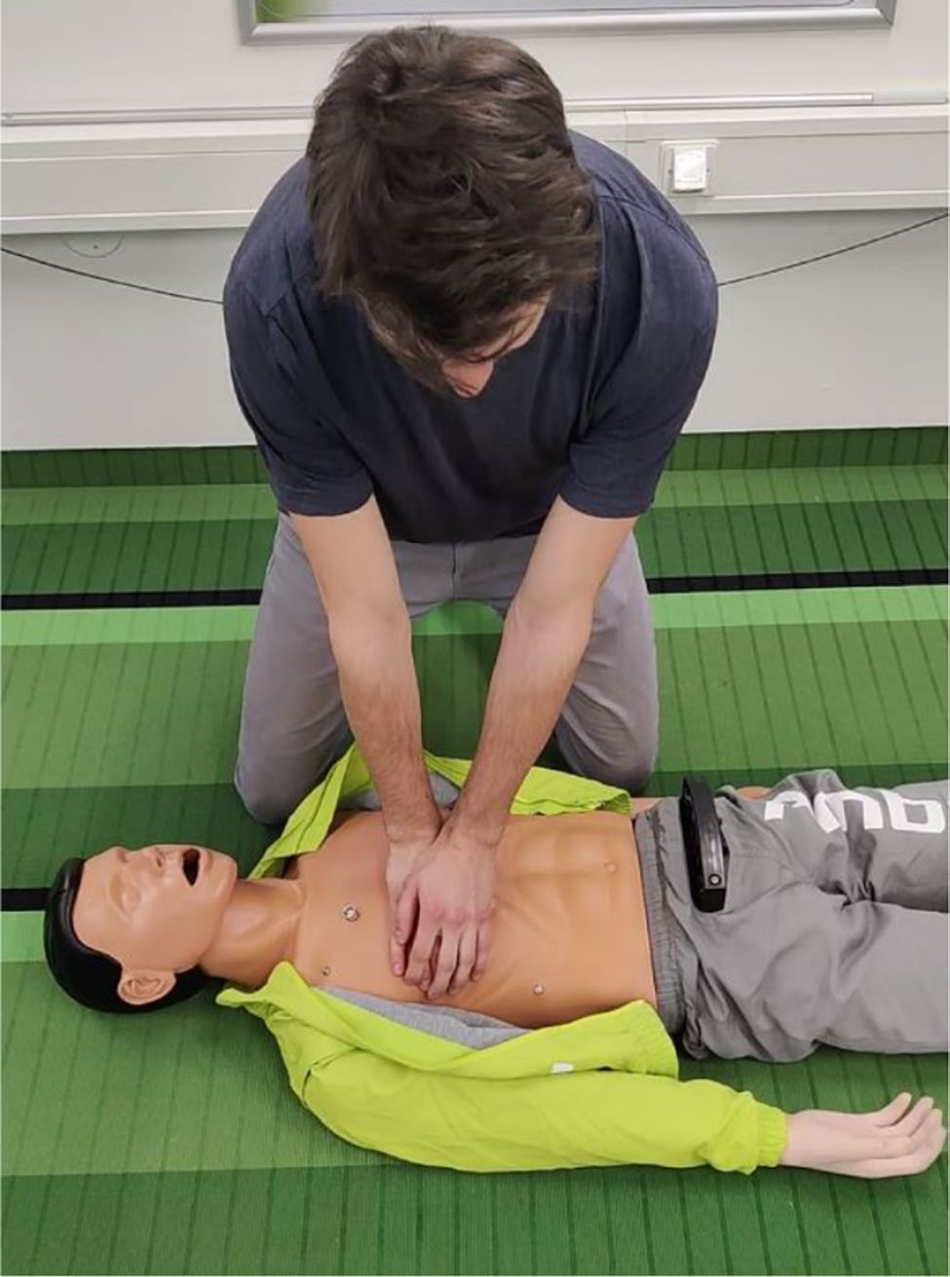
Screenshot of a video sequence used in the experiment

### Intervention

The data was gathered between March 18, 2024 and May 07, 2024, at Aachen University Hospital and the aforementioned rescue facilities. The demographic data was obtained using a standardized questionnaire (see supplements). During the experiment, participants evaluated video sequences of chest compressions performed on a manikin (Ambu Man wireless, Ambu GmbH, Bad Nauheim, Germany). The video sequences were presented on a notebook (HP 255 G10, HP Inc., Palo Alto, California) with a 15.6-inch screen and 1920 × 1080 pixels. Video sequences were recorded using a smartphone (OnePlus 8T, Oneplus, Shenzhen, China) with a resolution of 3840 × 2160 pixels per inch (8.3 megapixels) and 60 frames per second. In order to create a realistic situation, the smartphone was manually held at a distance of 140 centimetres from the manikin, at a height corresponding to the participants’ chest height and at an angle of 45 degrees. The manikin was positioned laterally, with the helper facing the camera (Fig. 2). A metronome (Metronom Beats App, Stonekick, London, UK; downloaded from the Google Play Store) was set to the appropriate frequency of 80, 110, and 140 beats per minute, respectively. The use of the compression depth indicator of the manikin ensured sufficient compression depth. In the final video sequences used in the experiment, the metronome was inaudible, and the compression depth display was not visible.

As an intervention, participants in the training group received a standardized training program on the recognition of too deep chest compressions prior to the evaluation process. This training program consisted of a standardized four-minute PowerPoint presentation (Microsoft PowerPoint, Microsoft Corporation, Washington, USA) that demonstrated five video sequences of correctly performed chest compressions and too deep compression depth, available in the RWTH Publications Repository (10.18154/RWTH-2025-00315). The compressions were performed by two male individuals in an indoor setting and under optimal lighting conditions. Participants in the control group did not receive any training.

**Fig. 3:**
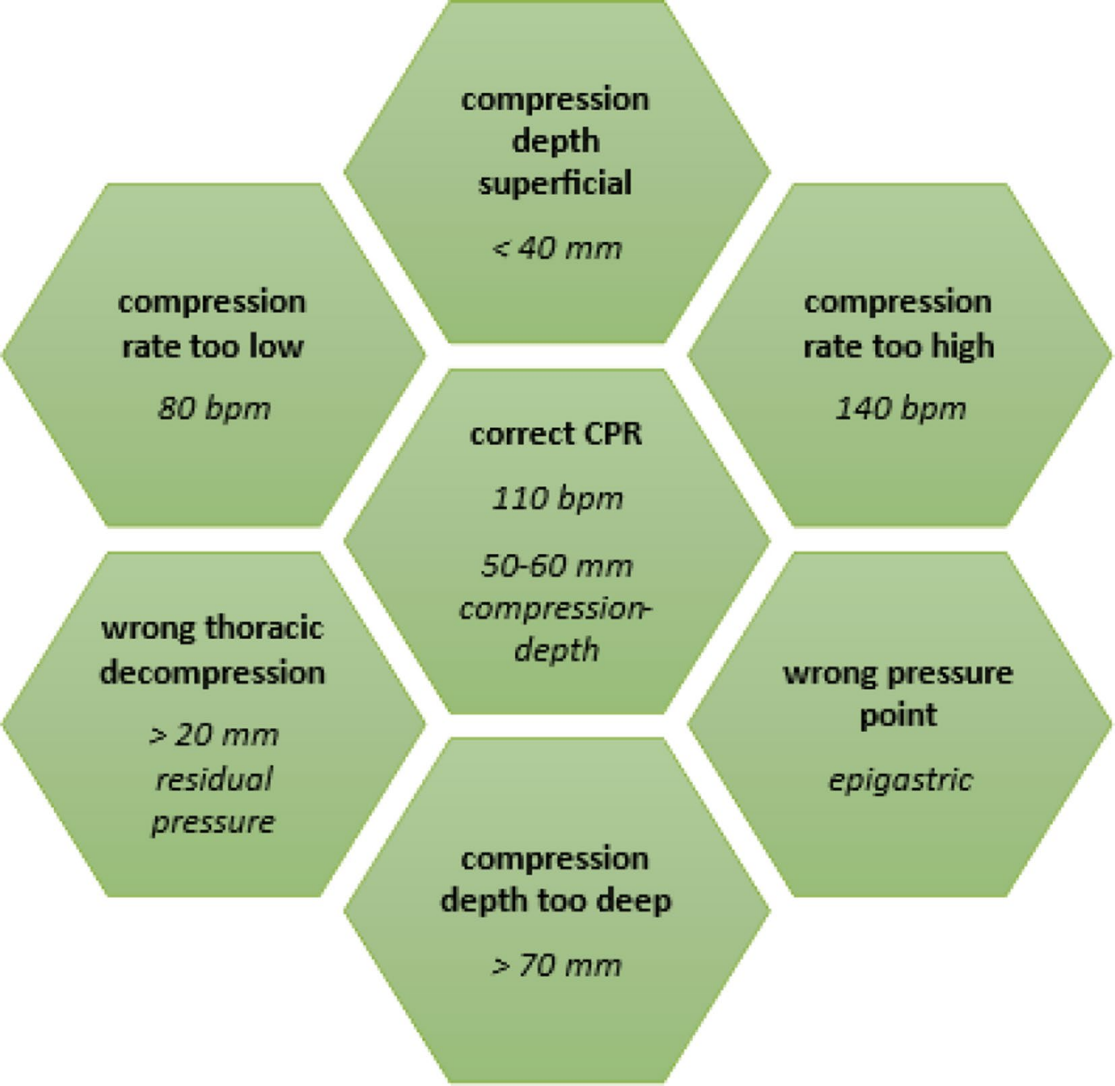
CPR parameters shown in the video sequences during the evaluation process

The video sequences presented during the subsequent evaluation phase were conducted in disparate settings by six individuals (four male, two female), including the same individuals who participated in the training videos. However, their demonstrated CPR performance errors differed. Each individual demonstrated seven scenarios of chest compressions, comprising one scenario of correctly performed compressions and six scenarios of typical errors during CPR (Fig. 3). Each sequence was ten seconds in duration and depicted only one single mistake. We have deliberately chosen this short duration for the videos in order to reflect the reality of everyday emergency dispatch center life, in which quick decisions are necessary. Video sequences used in the evaluation process differed from those used in the training process.

During the evaluation process, participants from both study groups evaluated 6 × 7 = 42 video sequences within a standardized PowerPoint presentation. Participants evaluated one video sequence after another with the help of a standardized questionnaire providing multiple choice answers on four main categories: compression rate (too high, correct, too low), pressure point (incorrect, correct), compression depth (superficial, correct, too deep), and thoracic decompression (incorrect, correct) (see supplements). The participants were informed that each video sequence contained only one error. Following the presentation of each video, the participants were permitted to take as much time as they required to select their response. It was not possible, however, to return to a previous video. The training and evaluation were conducted on the same laptop, utilising a brief introduction to familiarise the participants with the process.

### Outcomes

The primary endpoint focused on the rate of correct identification of chest compressions of too deep compression depth depending on the participants’ previous training. As secondary outcome parameters, we assessed the precision of classification of other presented errors and the presence of additional errors not evident in the video. Furthermore, we investigated the receptiveness of both study groups to integrating video instructions into daily work prior to evaluating the video sequences using a verbal rating scale with five levels: “not at all”, “a little”, “moderate”, “quite” and “very”. In the experimental group, we evaluated the effect of the short training intervention on participants’ confidence in assessing simulated CPR performance by employing the same five-point verbal rating scale. Therefore, the experimental group was surveyed before and after the training. The data set and software are available in the repository (10.18154/RWTH-2025-00315).

### Statistical analysis

The sample size calculation was based on previous studies [19, 20] with a power level set at 90% and a adjusted level of significance set at 5%. It was hypothesized that the intervention would increase the percentage of correct classification of chest compressions with too deep compression depth of more than 50%. The sample size was calculated with G*Power [22], version 3.1.9.6. For a Wilcoxon-Mann-Whitney test for two independent samples (two-tailed, alpha = 0.05, power 1-beta = 0.90, Cohen’s d = 0.8), a total *n* = 72 (2 × 36) was required. To account for an expected dropout of ~ 20%, we plannd with a total n of 90 with 45 participants in each study group. For the analysis of the primary endpoint, the Mann-Whitney-U-test was performed. Data of the secondary endpoints including the participants’ questionnaire responses regarding potential parameters influencing the ability to recognize errors were analysed descriptively, with normally distributed data presented as mean ± standard deviation (SD). For the secondary endpoint, a chi-square test was performed to determine whether there was a discrepancy in the detection rate of errors across all categories between the two study groups. To assess the performance of the untrained categories individually, a Mann-Whitney-U-test was conducted. Given the execution of multiple Mann-Whitney-U-tests to compare seven categories, a Bonferroni correction was used by dividing the original significance level of α = 0.05 by the number of tests conducted (7 tests). The corrected p-values were then compared to this new significance threshold of α = 0.007 [23]. A dependent two-tailed t-test was utilized to assess the pre- and post-training confidence levels of the participants. Additionally, we evaluated the participants’ openness to incorporating video-assisted CPR into their routine clinical practice.

## Results

### Characteristics of study participants

Enrollment was concluded once the predetermined number of cases had been attained. In total, *n* = 86 paramedics and *n* = 4 emergency physicians were recruited. All of the participants worked as paramedics or emergency physicians and only a few of them also worked in the dispatch center. Due to an emergency call, two participants (EMS) did not finish the experiment and were excluded. Finally, *N* = 88 participants were included and analysed. Participants’ characteristics are listed in Table 1.

**Table 1:** Descriptive overview of participants’ characteristics

	Control group	Experimental group
Sex		
Female [n]	12 (27.3%)	13 (29.5%)
Male [n]	32 (72.7%)	31 (70.5%)
Age [years]	31.8 ± 9.6	29.8 ± 8.1
Medical profession		
Paramedics [n]	42 (95.5%)	42 (95.5%)
Emergency physicians [n]	2 (4.5%)	2 (4.5%)
Professional experience [years]	9.6 ± 8.7	7.7 ± 6.9

### Primary endpoint

In total, 3696 video sequences were evaluated, thereof 528 video sequences of each parameter (Fig. 3) with a total of 14,784 requested response marks. In five videos, probands marked more than one answer within a single category (0.14%). As a result, those answers could not be analysed and were subsequently excluded. Further, participants of both study groups did not mark any answer in 14 videos (0.38%), four (0.11%) in the experimental group and 10 (0.27%) in the control group, leading to exclusion of the corresponding answers. Finally, 30 times (0.20%) in the experimental group and 71 times (0.48%) in the control group, a category was marked as “not assessable” leading to exclusion of the corresponding answers. Although participants had been informed that each video would display only a single error, *n* = 11 subjects marked more than one mistake in different categories at the same time in at least one of the 42 videos (experimental group: 4, control group: 7).

**Fig. 4:**
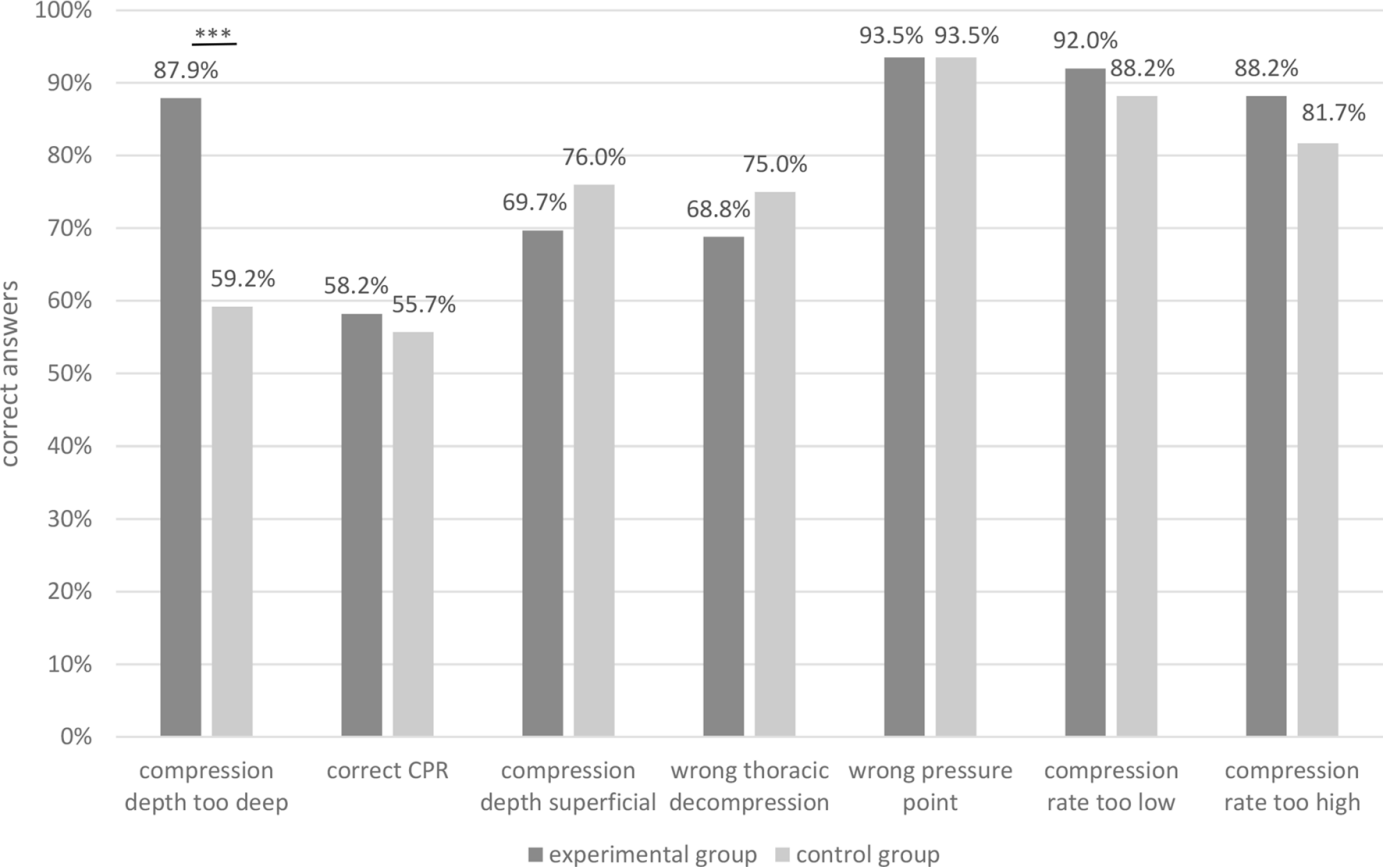
Correct classification of CPR errors depending on the previous training, ***=significant difference

The frequencies of the video sequences that were correctly classified are presented in Fig. 4. Thereby, a significant difference between the study groups was found regarding the correct classification of too deep chest compressions. Members of the experimental group correctly evaluated 232 video sequences of too deep compressions, while 155 in the control group were analysed correctly (87,9% vs. 59,2%, *p* < 0.001). Regarding the other mistakes, there were no significant differences between the study groups (*p*_correct_CPR_ = 0.598, *p*_compression_depth_superficial_ = 0.118, *p*_wrong_thoracic_decompression_ = 0.179, *p*_wrong_pressure_point_ = 1.0, *p*_compression_rate_too_low_ = 0.111, *p*_compression_rate_too_high_ = 0.28).

### Secondary endpoints

Overall, 2861 of 3682 videos (77,7%) were correctly classified [experimental group: 1471 of 1844 (79,8%); control group: 1390 of 1838 (75,6%)], showing a significant difference between the study groups (*p* = 0.003). In Table 2, the frequencies of correct classifications are presented depending on the presented errors and the study groups. Additionally, the frequencies and distribution of incorrectly classified video sequences are shown.

**Table 2:** Frequencies of classified errors depending on the presented error in a video sequence. Correct answers are indicated by bold font

			Answers given by the participants						
			Compression depth too deep	Correct CPR	Compression depth superficial	Wrong thoracic decompression	Wrong pressure point	Compression rate too low	Compression rate too high
CPR parameters shown in the video sequences	Compression depth too deep	Experimental group	**87.9%**	7.6%	0.4%	2.3%	0.0%	0.0%	3.0%
		Control group	**59.2%**	25.2%	0.8%	6.5%	3.1%	2.7%	5.3%
	Correct CPR	Experimental group	6.8%	**58.2%**	11.8%	16.3%	2.3%	2.3%	0.4%
		Control group	1.5%	**55.7%**	14.4%	18.6%	5.3%	2.3%	0.8%
	Compression depth superficial	Experimental group	0.4%	11.0%	**69.7%**	12.5%	1.1%	6.1%	0.0%
		Control group	0.0%	4.9%	**76.0%**	13.7%	5.7%	4.2%	0.4%
	Wrong thoracic decompression	Experimental group	1.1%	2.7%	23.6%	**68.8%**	0.4%	4.6%	0.4%
		Control group	0.4%	0.4%	27.3%	**75.0%**	3.1%	3.1%	0.0%
	Wrong pressure point	Experimental group	6.5%	0.8%	0.4%	0.8%	**93.5%**	0.4%	0.4%
		Control group	4.9%	0.8%	2.7%	2.7%	**93.5%**	1.1%	0.4%
	Compression rate too low	experimental group	0.8%	4.9%	0.4%	0.0%	1.5%	**92.0%**	0.4%
		Control group	0.8%	8.0%	2.7%	2.3%	0.8%	**88.2%**	0.4%
	Compression rate too high	Experimental group	0.4%	3.8%	4.6%	4.6%	0.4%	0.4%	**88.2%**
		Control group	0.8%	2.3%	8.0%	11.8%	1.5%	0.8%	**81.7%**

#### Participants’ self-assessment

**Fig. 5:**
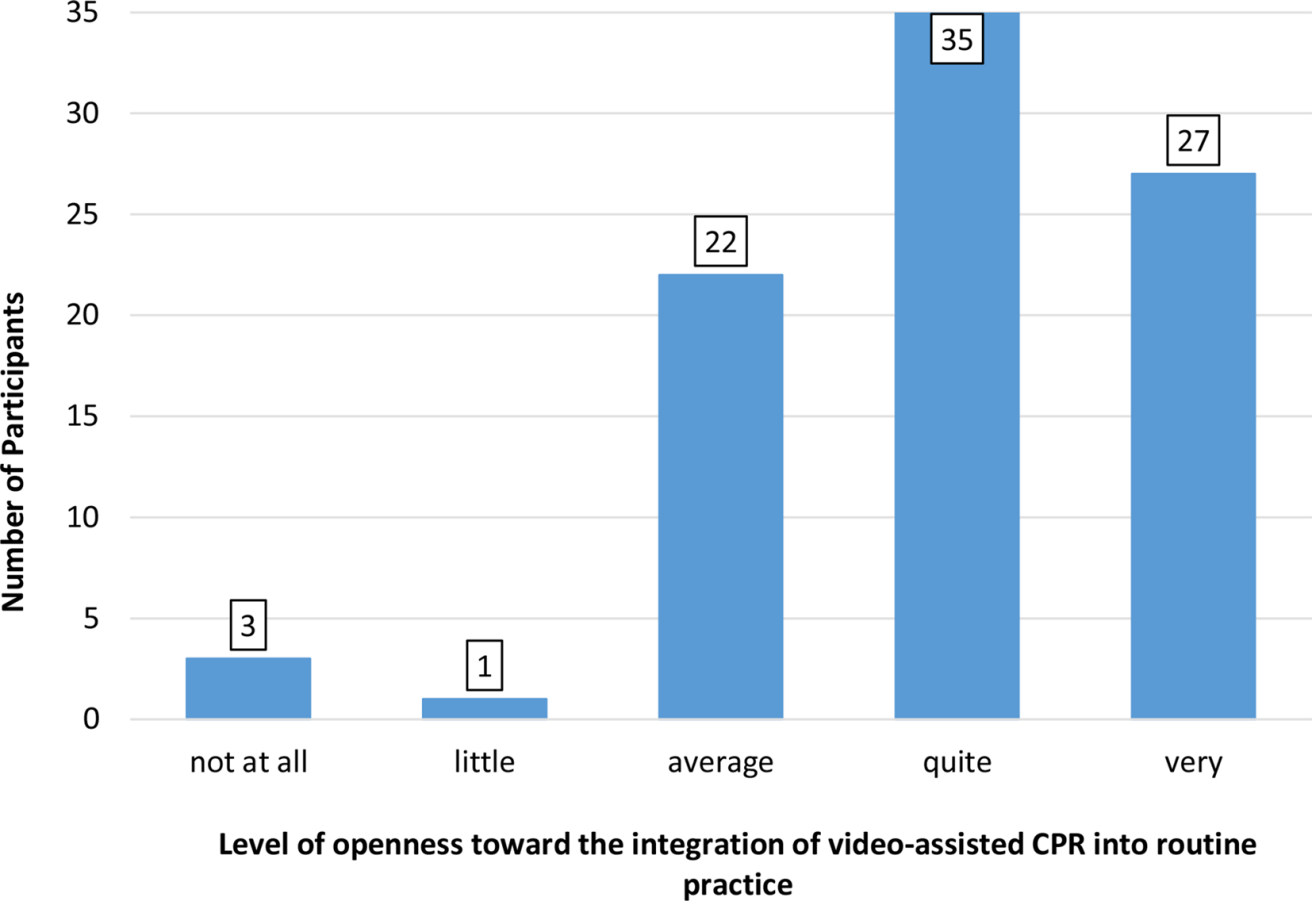
Frequency distribution of participants’ openness ratings towards the integration of video-assisted CPR into routine practice

**Fig. 6:**
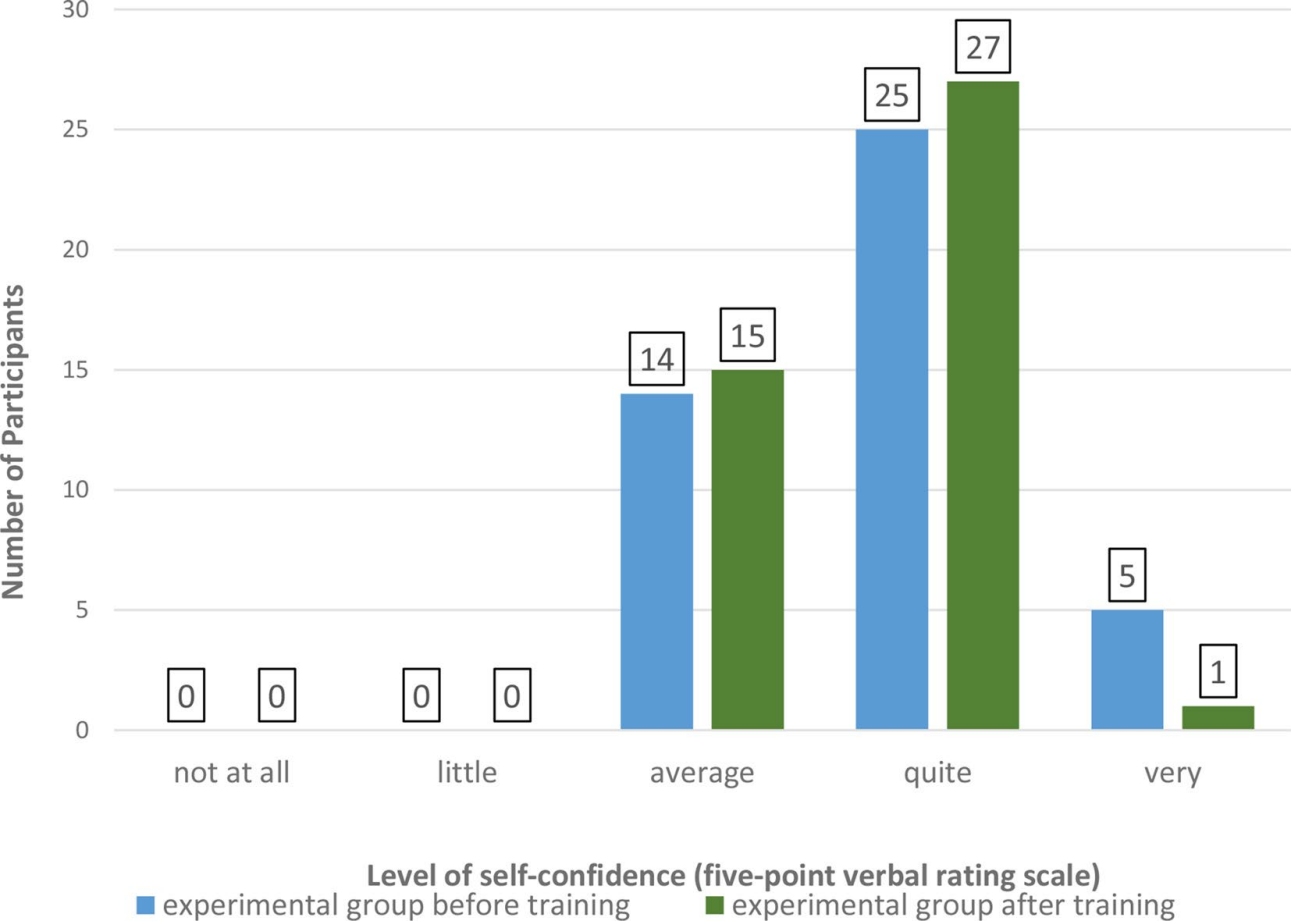
Frequency distribution of self-confidence levels before and after short video-CPR training, assessed using a five-point verbal rating scale

We investigated the attitudes of participants from both the experimental and control groups toward the integration of video instructions into routine professional practice, with the results presented in Fig. 5. In addition, we assessed the level of self-confidence in evaluating video-based CPR within the experimental group (*n* = 44), both before and after the training. The results are summarized in Fig. 6. No significant difference in self-confidence was observed in the experimental group before and after the short training (*p* = 0.44).

## Discussion

The findings of the present study demonstrate that a brief training intervention focused on identifying too deep chest compressions results in a substantial enhancement in the recognition rate of such compressions, displayed in a video sequence. Conversely, the training had no significant impact on the recognition rate of other errors observed during the evaluation process. Due to the training, evaluation rate of the experimental group was significantly elevated compared to the control group. Moreover, the majority of participants expressed openness to incorporating video-CPR into their daily work.

The objective of the brief training session was to illustrate and impart to the members of the experimental group an excessive compression depth. Interestingly, videos showing a correct CPR scenario were not recognized significantly more frequently compared to the other CPR scenarios, although videos showing a correct CPR were part of the training videos as a direct comparison to compressions with too deep compression depth. A correctly performed CPR sequence was accurately identified in only slightly more than half of the cases. This finding is consistent with previous studies examining the impact of video quality and camera perspective on the reliability of performance assessments [19, 20]. The limited ability to recognize adequate chest compressions is concerning, as it may lead to inappropriate corrective feedback, thereby inadvertently compromising the quality of resuscitation. Future training programs in video-based CPR assessment should therefore place particular emphasis on the recognition of correctly executed CPR. One possible explanation is that participants in the intervention group may have anticipated the presence of some kind of error in each video. This observation was reported by the participants themselves after completing the experiment. Another potential explanation could be found in the design of the evaluation form. When evaluating a video sequence, participants were tasked with assessing four categories: compression depth, compression rate, hand position, and thoracic decompression. To evaluate a correct CPR performance, participants were required to correctly rate all four categories. In contrast, the presented CPR errors were rated as correctly recognized when the single corresponding category was correctly marked. Participants may also have inferred the intended focus of the training video and, as a result, developed an expectation to specifically identify excessively deep chest compressions. This selective attention could have inadvertently shifted their perceptual standard, thereby impairing their sensitivity to other clinically relevant deviations. Although the differences did not reach statistical significance, participants in the control group more frequently identified insufficient compression depth and incomplete chest recoil, whereas participants in the experimental group did so correspondingly less often. These findings underscore the necessity of training formats that encompass a broader spectrum of potential errors. Addressing multiple high-risk deviations - rather than a single error category - may be essential to improve the accuracy and clinical utility of resuscitation assessments. 

The findings of the present study further demonstrate that the participants encountered challenges in differentiating between videos demonstrating superficial compression depth and wrong thoracic decompression. This observation is consistent with the results of a previous study conducted by the authors [20]. The difficulty in differentiating between these categories can be attributed to the fact that the amplitude of compression is equivalent in both scenarios. Despite being informed in advance that each video would contain a maximum of one error, few participants identified multiple errors concurrently. This finding suggests that participants may have been reluctant to make a subjective decision regarding the errors they recognized, opting instead for the most evident error, likely anticipating that a maximum of one error would be displayed. In future studies, either the possibility of giving multiple answers should be excluded (e.g., by using an electronic questionnaire), or the study design should be adapted to explicitly allow multiple responses. Following the conclusion of the experiment, certain participants offered constructive criticism regarding the lateral and rigid camera position. Previous studies have demonstrated that the filming perspective opposite the resuscitating individual is the one with the highest recognition rate [19]. On this basis, we have opted for the chosen perspective. To the best of our knowledge, the effects of dynamic camera guidance have not yet been thoroughly examined and described in the extant literature.

With respect to the evaluation of integrating video-assisted cardiopulmonary resuscitation during dispatcher assistance, the majority of participants expressed a willingness to incorporate video-assisted CPR into their daily work practices. However, there are several critical aspects that necessitate deliberation. Primarily, the individual responsible for documenting the event may be perceived as an external observer in a real-life context, where the phenomenon of rubbernecking is prevalent [24]. Therefore, filming of an emergency by a first responder for the purpose of providing better medical care could serve as a justification for gawkers to film themselves for sensationalism. It may be assumed that this issue would predominantly impact urban areas, particularly those with a high volume of passenger or vehicular traffic. Conversely, in less densely populated areas, where the distances ambulances travel are greater and telemedicine plays a more significant role [25], this phenomenon would presumably be of minimal consequence when weighed against the benefits. Furthermore, it can be argued that in cases where resuscitation efforts prove unsuccessful, relatives of the deceased are entitled to the assurance that every possible measure was taken to save their family member or friend [26]. Notwithstanding the potential for criticism enumerated above, it is noteworthy that Linderoth et al. found that 97.3% of the callers (first responders who made the emergency call) reported in favour of the implementation of live video in emergency calls in general [27]. In the context of video-assisted first aid measures, Idland et al. demonstrated that “the need for first aid measures was recognized more often during the medical emergency call when the dispatcher used video streaming”. The authors concluded that the use of video streaming may therefore increase the frequency of bystander first aid [28]. These findings can likely be extrapolated to video-assisted CPR, particularly concerning the challenges dispatchers face in accurately assessing cardiac arrest situations. In addition to identifying common misjudgements during video-assisted CPR [15, 16], the initial recognition of cardiac arrest via telephone-only is challenging for dispatchers to adequately assess [29, 30]. In contrast to telephone CPR, Linderoth et al. found that incorporating live video into a telephone call significantly influenced and improved the dispatcher’s assessment of the situation. Regarding the impact of video-assisted communication on the outcomes of patients who have suffered an out-of-hospital cardiac arrest, Lee et al. revealed that the survival to discharge rate and the proportion of patients with good neurological outcomes were significantly higher in the video group compared to the audio-only group [31]. As indicated by rescue centre staff regarding telephone-CPR [10] training courses and education are mandatory for the successful implementation of any approach. In the present study, we demonstrated that the selected training method, explicitly designed to improve the identification of excessively deep chest compressions, significantly enhances the detection rate of this specific error. Accordingly, this training approach has the potential to be extended and applied to other error types in video-based CPR assessment.

## Conclusion

The identification of chest compressions with too deep compression depth in video recordings of simulated CPR significantly improved among evaluators following a brief training intervention within the context of a simulation-based study. Further research is warranted to evaluate the effectiveness of training in enhancing the recognition of other common CPR performance errors.

### Limitations

First, the present study was based on the evaluation of video sequences depicting optimal circumstances, such as lightning conditions, a second helper holding the camera in an optimal position and only one error per video sequence. Second, CPR was provided on a training manikin and not on humans. Furthermore, the evaluation process was conducted in a quiet setting without competing tasks or stress factors. In real-life settings, the assessment of CPR performance based on video observation represents a continuous process, which is not limited - as it is in this study - to a duration of only 10 s. Moreover, since the evaluation of the videos was conducted immediately after the training, no conclusions can be drawn regarding the durability of the training effect. In summary, these laboratory conditions are not transferable to reality and certainly do not represent the complexity inherent to real-life CPR situations. Thus, results of this study need to be confirmed in real-life study settings.

## Electronic supplementary material

Below is the link to the electronic supplementary material.

Supplementary Material 1

Supplementary Material 2

## Data Availability

The datasets generated and analyzed during the current study, as well as the training video used in the study, are available in the RWTH Publications Repository: 10.18154/RWTH-2025-00315.

## Abbreviations

CPR: Cardiopulmonary resuscitation
EMS: Emergency medical service
EP: Emergency physician
OHCA: Out-of-hospital cardiac arrest
SD: Standard deviation
T-CPR: Telephone cardiopulmonary resuscitation
VA-CPR: Video-assisted cardiopulmonary resuscitation
