# Supplementary material for: Impact of a short training on the recognition of excessively deep chest compressions during video-assisted cardiopulmonary resuscitation: a randomized controlled simulation trial
Source: BMC Med Educ. 2025 Jul 11;25:1033. doi: 10.1186/s12909-025-07524-w (PMC12247412; doi:10.1186/s12909-025-07524-w)
Supplement: Supplementary file 1 — Supplementary Material 1 [file 12909_2025_7524_MOESM1_ESM.docx]

Questionnaire for participation in the research project

*Impact of a short training when evaluating video-assisted cardiopulmonary resuscitation: a randomized, controlled simulation trial*

Dear participant,

as part of your participation in the above-mentioned study, we kindly ask you to complete the following questionnaire. Thank you very much!

Randomisation number (is entered by the study staff):_________________

1. Which gender do you identify with?

M F D

1. How old are you? _________ Years
2. Which medical profession do you belong to?

Paramedics E Emergency physician

1. How many years of professional experience do you have? ______________ Years
2. How open are you to integrate video instructions from lay first aiders into your daily work?

not at all a little average quite very

1. How confident do you feel when assessing a resuscitation you have observed?

not at all a little average quite very

Date and time (entered by the study staff):
