# Supplementary material for: Impact of a short training on the recognition of excessively deep chest compressions during video-assisted cardiopulmonary resuscitation: a randomized controlled simulation trial
Source: BMC Med Educ. 2025 Jul 11;25:1033. doi: 10.1186/s12909-025-07524-w (PMC12247412; doi:10.1186/s12909-025-07524-w)
Supplement: Supplementary file 2 — Supplementary Material 2 [file 12909_2025_7524_MOESM2_ESM.docx]

**Questionnaire for study participants**

*Impact of a short training when evaluating video-assisted cardiopulmonary resuscitation: a randomized, controlled simulation trial*

Dear participant,

you will be shown 42 different video sequences of simulated resuscitation. We ask you to assess the quality of resuscitation based on the criteria of compression depth, compression rate, pressure point and thoracic decompression. If one or more criteria are not assessable from your point of view, tick this option. It is not possible to repeat a video, return to a previous question or correct an answer that has already been given.

If you have taken part in the short training course, please indicate again how confident you feel subjectively regarding the assessment of a video CPR.

Thank you very much!

How confident do you feel when assessing a resuscitation? (Please only complete if you have taken part in the training course!)

not at all a little average quite very

**Video 1:** *Compression rate* □ too low □ correct □ too high □ not assessable
*Pressure point* □ wrong □ correct □ not assessable
*Compression depth* □ superficial □ correct □ too deep □ not assessable
*Thoracic decompression* □ wrong □ correct □ not assessable

**Video 2:** *Compression rate* □ too low □ correct □ too high □ not assessable
*Pressure point* □ wrong □ correct □ not assessable
*Compression depth* □ superficial □ correct □ too deep □ not assessable
*Thoracic decompression* □ wrong □ correct □ not assessable

**Video 3:** *Compression rate* □ too low □ correct □ too high □ not assessable
*Pressure point* □ wrong □ correct □ not assessable
*Compression depth* □ superficial □ correct □ too deep □ not assessable
*Thoracic decompression* □ wrong □ correct □ not assessable

**Video 4:** *Compression rate* □ too low □ correct □ too high □ not assessable
*Pressure point* □ wrong □ correct □ not assessable
*Compression depth* □ superficial □ correct □ too deep □ not assessable
*Thoracic decompression* □ wrong □ correct □ not assessable

**Video 5:** *Compression rate* □ too low □ correct □ too high □ not assessable
*Pressure point* □ wrong □ correct □ not assessable
*Compression depth* □ superficial □ correct □ too deep □ not assessable
*Thoracic decompression* □ wrong □ correct □ not assessable

**Video 6:** *Compression rate* □ too low □ correct □ too high □ not assessable
*Pressure point* □ wrong □ correct □ not assessable
*Compression depth* □ superficial □ correct □ too deep □ not assessable
*Thoracic decompression* □ wrong □ correct □ not assessable

**Video 7:** *Compression rate* □ too low □ correct □ too high □ not assessable
*Pressure point* □ wrong □ correct □ not assessable
*Compression depth* □ superficial □ correct □ too deep □ not assessable
*Thoracic decompression* □ wrong □ correct □ not assessable

**Video 8:** *Compression rate* □ too low □ correct □ too high □ not assessable
*Pressure point* □ wrong □ correct □ not assessable
*Compression depth* □ superficial □ correct □ too deep □ not assessable
*Thoracic decompression* □ wrong □ correct □ not assessable

**Video 9:** *Compression rate* □ too low □ correct □ too high □ not assessable
*Pressure point* □ wrong □ correct □ not assessable
*Compression depth* □ superficial □ correct □ too deep □ not assessable
*Thoracic decompression* □ wrong □ correct □ not assessable

**Video 10:** *Compression rate* □ too low □ correct □ too high □ not assessable
*Pressure point* □ wrong □ correct □ not assessable
*Compression depth* □ superficial □ correct □ too deep □ not assessable
*Thoracic decompression* □ wrong □ correct □ not assessable

**Video 11:** *Compression rate* □ too low □ correct □ too high □ not assessable
*Pressure point* □ wrong □ correct □ not assessable
*Compression depth* □ superficial □ correct □ too deep □ not assessable
*Thoracic decompression* □ wrong □ correct □ not assessable

**Video 12:** *Compression rate* □ too low □ correct □ too high □ not assessable
*Pressure point* □ wrong □ correct □ not assessable
*Compression depth* □ superficial □ correct □ too deep □ not assessable
*Thoracic decompression* □ wrong □ correct □ not assessable

**Video 13:** *Compression rate* □ too low □ correct □ too high □ not assessable
*Pressure point* □ wrong □ correct □ not assessable
*Compression depth* □ superficial □ correct □ too deep □ not assessable
*Thoracic decompression* □ wrong □ correct □ not assessable

**Video 14:** *Compression rate* □ too low □ correct □ too high □ not assessable
*Pressure point* □ wrong □ correct □ not assessable
*Compression depth* □ superficial □ correct □ too deep □ not assessable
*Thoracic decompression* □ wrong □ correct □ not assessable

**Video 15:** *Compression rate* □ too low □ correct □ too high □ not assessable
*Pressure point* □ wrong □ correct □ not assessable
*Compression depth* □ superficial □ correct □ too deep □ not assessable
*Thoracic decompression* □ wrong □ correct □ not assessable

**Video 16:** *Compression rate* □ too low □ correct □ too high □ not assessable
*Pressure point* □ wrong □ correct □ not assessable
*Compression depth* □ superficial □ correct □ too deep □ not assessable
*Thoracic decompression* □ wrong □ correct □ not assessable

**Video 17:** *Compression rate* □ too low □ correct □ too high □ not assessable
*Pressure point* □ wrong □ correct □ not assessable
*Compression depth* □ superficial □ correct □ too deep □ not assessable
*Thoracic decompression* □ wrong □ correct □ not assessable

**Video 18:** *Compression rate* □ too low □ correct □ too high □ not assessable
*Pressure point* □ wrong □ correct □ not assessable
*Compression depth* □ superficial □ correct □ too deep □ not assessable
*Thoracic decompression* □ wrong □ correct □ not assessable

**Video 19:** *Compression rate* □ too low □ correct □ too high □ not assessable
*Pressure point* □ wrong □ correct □ not assessable
*Compression depth* □ superficial □ correct □ too deep □ not assessable
*Thoracic decompression* □ wrong □ correct □ not assessable

**Video 20:** *Compression rate* □ too low □ correct □ too high □ not assessable
*Pressure point* □ wrong □ correct □ not assessable
*Compression depth* □ superficial □ correct □ too deep □ not assessable
*Thoracic decompression* □ wrong □ correct □ not assessable

**Video 21:** *Compression rate* □ too low □ correct □ too high □ not assessable
*Pressure point* □ wrong □ correct □ not assessable
*Compression depth* □ superficial □ correct □ too deep □ not assessable
*Thoracic decompression* □ wrong □ correct □ not assessable

**Video 22:** *Compression rate* □ too low □ correct □ too high □ not assessable
*Pressure point* □ wrong □ correct □ not assessable
*Compression depth* □ superficial □ correct □ too deep □ not assessable
*Thoracic decompression* □ wrong □ correct □ not assessable

**Video 23:** *Compression rate* □ too low □ correct □ too high □ not assessable
*Pressure point* □ wrong □ correct □ not assessable
*Compression depth* □ superficial □ correct □ too deep □ not assessable
*Thoracic decompression* □ wrong □ correct □ not assessable

**Video 24:** *Compression rate* □ too low □ correct □ too high □ not assessable
*Pressure point* □ wrong □ correct □ not assessable
*Compression depth* □ superficial □ correct □ too deep □ not assessable
*Thoracic decompression* □ wrong □ correct □ not assessable

**Video 25:** *Compression rate* □ too low □ correct □ too high □ not assessable
*Pressure point* □ wrong □ correct □ not assessable
*Compression depth* □ superficial □ correct □ too deep □ not assessable
*Thoracic decompression* □ wrong □ correct □ not assessable

**Video 26:** *Compression rate* □ too low □ correct □ too high □ not assessable
*Pressure point* □ wrong □ correct □ not assessable
*Compression depth* □ superficial □ correct □ too deep □ not assessable
*Thoracic decompression* □ wrong □ correct □ not assessable

**Video 27:** *Compression rate* □ too low □ correct □ too high □ not assessable
*Pressure point* □ wrong □ correct □ not assessable
*Compression depth* □ superficial □ correct □ too deep □ not assessable
*Thoracic decompression* □ wrong □ correct □ not assessable

**Video 28:** *Compression rate* □ too low □ correct □ too high □ not assessable
*Pressure point* □ wrong □ correct □ not assessable
*Compression depth* □ superficial □ correct □ too deep □ not assessable
*Thoracic decompression* □ wrong □ correct □ not assessable

**Video 29:** *Compression rate* □ too low □ correct □ too high □ not assessable
*Pressure point* □ wrong □ correct □ not assessable
*Compression depth* □ superficial □ correct □ too deep □ not assessable
*Thoracic decompression* □ wrong □ correct □ not assessable

**Video 30:** *Compression rate* □ too low □ correct □ too high □ not assessable
*Pressure point* □ wrong □ correct □ not assessable
*Compression depth* □ superficial □ correct □ too deep □ not assessable
*Thoracic decompression* □ wrong □ correct □ not assessable

**Video 31:** *Compression rate* □ too low □ correct □ too high □ not assessable
*Pressure point* □ wrong □ correct □ not assessable
*Compression depth* □ superficial □ correct □ too deep □ not assessable
*Thoracic decompression* □ wrong □ correct □ not assessable

**Video 32:** *Compression rate* □ too low □ correct □ too high □ not assessable
*Pressure point* □ wrong □ correct □ not assessable
*Compression depth* □ superficial □ correct □ too deep □ not assessable
*Thoracic decompression* □ wrong □ correct □ not assessable

**Video 33:** *Compression rate* □ too low □ correct □ too high □ not assessable
*Pressure point* □ wrong □ correct □ not assessable
*Compression depth* □ superficial □ correct □ too deep □ not assessable
*Thoracic decompression* □ wrong □ correct □ not assessable

**Video 34:** *Compression rate* □ too low □ correct □ too high □ not assessable
*Pressure point* □ wrong □ correct □ not assessable
*Compression depth* □ superficial □ correct □ too deep □ not assessable
*Thoracic decompression* □ wrong □ correct □ not assessable

**Video 35:** *Compression rate* □ too low □ correct □ too high □ not assessable
*Pressure point* □ wrong □ correct □ not assessable
*Compression depth* □ superficial □ correct □ too deep □ not assessable
*Thoracic decompression* □ wrong □ correct □ not assessable

**Video 36:** *Compression rate* □ too low □ correct □ too high □ not assessable
*Pressure point* □ wrong □ correct □ not assessable
*Compression depth* □ superficial □ correct □ too deep □ not assessable
*Thoracic decompression* □ wrong □ correct □ not assessable

**Video 37:** *Compression rate* □ too low □ correct □ too high □ not assessable
*Pressure point* □ wrong □ correct □ not assessable
*Compression depth* □ superficial □ correct □ too deep □ not assessable
*Thoracic decompression* □ wrong □ correct □ not assessable

**Video 38:** *Compression rate* □ too low □ correct □ too high □ not assessable
*Pressure point* □ wrong □ correct □ not assessable
*Compression depth* □ superficial □ correct □ too deep □ not assessable
*Thoracic decompression* □ wrong □ correct □ not assessable

**Video 39:** *Compression rate* □ too low □ correct □ too high □ not assessable
*Pressure point* □ wrong □ correct □ not assessable
*Compression depth* □ superficial □ correct □ too deep □ not assessable
*Thoracic decompression* □ wrong □ correct □ not assessable

**Video 40:** *Compression rate* □ too low □ correct □ too high □ not assessable
*Pressure point* □ wrong □ correct □ not assessable
*Compression depth* □ superficial □ correct □ too deep □ not assessable
*Thoracic decompression* □ wrong □ correct □ not assessable

**Video 41:** *Compression rate* □ too low □ correct □ too high □ not assessable
*Pressure point* □ wrong □ correct □ not assessable
*Compression depth* □ superficial □ correct □ too deep □ not assessable
*Thoracic decompression* □ wrong □ correct □ not assessable

**Video 42:** *Compression rate* □ too low □ correct □ too high □ not assessable
*Pressure point* □ wrong □ correct □ not assessable
*Compression depth* □ superficial □ correct □ too deep □ not assessable
*Thoracic decompression* □ wrong □ correct □ not assessable
